# Allelopathic and Bloom-Forming Picocyanobacteria in a Changing World

**DOI:** 10.3390/toxins10010048

**Published:** 2018-01-20

**Authors:** Sylwia Śliwińska-Wilczewska, Jakub Maculewicz, Aldo Barreiro Felpeto, Adam Latała

**Affiliations:** 1 Institute of Oceanography, Faculty of Oceanography and Geography, University of Gdansk, Av. Pilsudskiego 46, 81-378 Gdynia, Poland; kubeslaw@gmail.com (J.M.); oceal@univ.gda.pl (A.L.); 2 Interdisciplinary Center of Marine and Environmental Research–CIMAR/CIIMAR, University of Porto, Av. General Norton de Matos s/n, 4450-208 Matosinhos, Portugal; aldo.barreiro@gmail.com

**Keywords:** allelopathy, allelochemicals, climate change, cyanotoxins, picocyanobacteria, picoplankton, blooms, secondary metabolites, We have updated a review of the literature dealing with allelopathic activity of picocyanobacteria; their toxicity; occurrence of their massive blooms and the relationships between climate change and representative picocyanobacterial genera from aquatic ecosystems.

## Abstract

Picocyanobacteria are extremely important organisms in the world’s oceans and freshwater ecosystems. They play an essential role in primary production and their domination in phytoplankton biomass is common in both oligotrophic and eutrophic waters. Their role is expected to become even more relevant with the effect of climate change. However, this group of photoautotrophic organisms still remains insufficiently recognized. Only a few works have focused in detail on the occurrence of massive blooms of picocyanobacteria, their toxicity and allelopathic activity. Filling the gap in our knowledge about the mechanisms involved in the proliferation of these organisms could provide a better understanding of aquatic environments. In this review, we gathered and described recent information about allelopathic activity of picocyanobacteria and occurrence of their massive blooms in many aquatic ecosystems. We also examined the relationships between climate change and representative picocyanobacterial genera from freshwater, brackish and marine ecosystems. This work emphasizes the importance of studying the smallest picoplanktonic fractions of cyanobacteria.

## 1. Introduction

Picocyanobacteria (with cell size in the range of 0.2–2.0 μm) have been recognized in the last few years as important components of the phytoplankton not only in freshwaters and brackish ecosystems but also in the world ocean [1,2,3], constituting an important link in the food web and the basis of primary production [4]. Despite its small size, picocyanobacteria may account for up to 50% of cyanobacterial biomass in the world ocean [4]. They can be also responsible for up to 80–90% of the total carbon production in aquatic habitats [5]. 

Picoplanktonic cyanobacteria have developed many adaptations which enable them to spread in aquatic environments. What is more, picocyanobacteria often dominate and occupy the niches which are inaccessible for other photoautotrophs. Picocyanobacteria can float effectively in the water despite the absence of gas vacuoles which results directly from their extremely small size. Due to its small size, they also have a high surface to volume relationship. This enables a faster rate of nutrient uptake and hence growth rate, compared to larger phytoplankton cells [6]. Moreover, there are reports of unicellular picocyanobacterium *Synechococcus* sp. being able to fix atmospheric nitrogen [7]. Therefore, in oligotrophic regions of seas and oceans [3] as well as in the eutrophic basins and freshwater reservoirs [1,2] picocyanobacteria strong competitors in the phytoplankton community and this allows them to constitute the major fraction of primary production in worlds aquatic ecosystems [8].

Picoplanktonic cyanobacteria are also characterized by high consumer pressure [9,10]. Due to the small size, picocyanobacteria are a major food source for nanoplanktonic protozoa and larger zooplanktonic organisms [11]. Moreover, Motwani and Gorokhova [12] noted that copepods, cladocerans and rotifers were found to consume picocyanobacteria in substantial quantities and confirmed that copepod *Acartia tonsa* ingested *Synechococcus bacillaris*, even when the alternative food was plentiful. According to reports by Sorokin et al. [13], in some situations picocyanobacteria can provide up to 98% of phytoplankton production. By making such a large proportion of phytoplankton, picoplanktonic organisms affect not only the composition and quantity of matter but also the flow of energy to higher trophic levels [14]. Furthermore, Motwani and Gorokhova [12] suggested that, picoplankton are important components of mesozooplankton diet, which needs to be taken into account in food web models and productivity assessments. In surface layers, the number of picocyanobacteria varies from a few hundred to several thousand and sometimes even a few million cells per mL of water [14,15]. The large number of autotrophic picoplankton makes these organisms crucial for the ecological stability of aquatic ecosystems [16].

## 2. The Significance of Picocyanobacteria in Response to Global Change

Global changes in the ocean in the twenty-first century include warming waters, increased water stratification, altered light environments, increased CO_2_ and lower pH [17]. Additionally, due to other human activities such as agriculture, there is an increased eutrophication, particularly in coastal areas. Then, phytoplankton organisms, including picocyanobacteria, are currently facing multiple environmental changes [17]. Under certain environmental conditions, populations of some species of picocyanobacteria may achieve high abundances and, in consequence, adversely affect other organisms by producing harmful secondary metabolites or contributing to the development of anaerobic conditions in the environment. This phenomenon, called massive blooms, can be caused by both marine and freshwater species of picocyanobacteria [18]. 

In recent decades, the incidence and intensity of cyanobacterial harmful blooms has increased in aquatic ecosystems [19]. In addition, Brutemark et al. [20] and Paerl and Huisman [21] noted that some bloom-forming cyanobacteria will probably get more common in the future, due to their ability to produce and release allelopathic compounds and because of climate changes. Currently it is believed that cyanobacterial blooms are complex events caused by multiple factors occurring simultaneously [19]. Therefore, detailed studies are needed to fully clarify which environmental factors may influence massive picocyanobacterial occurrence.

Irradiance is one of the major factors controlling growth, photosynthetic activity and distribution of picocyanobacteria [10,22]. Cyanobacteria are generally recognized to prefer low light intensity for growth [23]. Some literature data indicated that under culturing conditions, some strains of picoplanktonic photoautotrophs showed survival and resurgence after 24 weeks of total darkness [24]. Such a pronounced capacity for survival in the dark would enable these organisms to outlive the seasonal rhythm of winter darkness and sinking into the aphotic zone [25]. On the other hand, Kana and Glibert [26] showed that marine *Synechococcus* sp. could grow at irradiance as high as 2000 μmol photons m^−2^ s^−1^. The experiments on three Baltic *Synechococcus* strains demonstrated their tolerance to elevated light levels and their high capacity to acclimate to irradiance [22]. These strains were able to change the composition of photosynthetic pigments to use light quanta better and to protect themselves from unfavourable effect of excessive light. The ability of *Synechococcus* to sustain their growth rate in low light conditions and their potentially low photoinhibition in exposure to high light intensities could give picocyanobacteria an advantage in changeable light-limited waters. This also explains why Baltic *Synechococcus* sp. grow successfully in both well-illuminated surface waters and deeper waters [27]. Irradiance could also play an important role in the regulation of allelochemical production in some picocyanobacteria species [28,29], thus this factor in response to global change, should be considered a significant driving force in sustaining picocyanobacterial blooms.

Temperature is also a very important driver of picocyanobacteria growth and abundance. Significant relationships between picocyanobacteriagrowth rates and biomass accrual have been reported by a number of authors working on a variety of systems (e.g., [30,31]). It has been also shown that an increase in surface water temperatures due to changing global climate could play a role in the proliferation of cyanobacterial blooms [32,33]. In the current century, global air temperatures are expected to increase by additional 1.5–5 °C [34]. Paerl and Huisman [33] noted that the global temperature rise would stabilize or inhibit the eukaryotic phytoplankton, while the number of cyanobacteria would increase. Regarding climate change, picocyanobacteria achieves maximal growth rates at higher temperatures than other cyanobacteria [35] and thus will potentially be promoted by future climatic warming. In laboratory studies, Jodłowska and Śliwińska [22] also found that increasing temperatures from 15 °C to 30 °C increased picocyanobacterial abundances. In addition, Śliwińska-Wilczewska et al. [28] examined whether the production of allelopathic substances by picocyanobacteria is regulated by temperature. The sum of research conducted regarding the ecophysiology and in situ dynamics of picocyanobacteria suggests that they will thrive under the conditions predicted for global climate change [32,33]. The details of how specific genera of picocyanobacteria may respond to climate change are less clear and require further detailed research.

Ocean acidification is another impact of climate change that was suggested to result in a relative increase of picocyanobacteria in ocean phytoplankton communities [17] but so far, there is no supporting evidence from field mesocosms experiments [36].

## 3. Morphological and Physiological Characteristics of Picoplanktonic Cyanobacteria

Picocyanobacteria are ecologically and genetically diverse and include many clads and species [37]. Picoplanktonic cyanobacteria are usually single-celled forms but may also appear in microcolonies [1]. In marine water, unicellular picoplankton is most often represented by organisms of the genus *Prochlorococcus* and *Synechococcus*. In freshwaters ecosystems, the diversity of single-celled picocyanobacteria is greater and includes such genera as *Synechococcus*, *Cyanobium*, *Synechocystis*, *Cyanothece* and *Cyanobacterium* [1]. Moreover, colonial picocyanobacteria in freshwater habitats are species of *Aphanocapsa*, *Aphanothece*, *Chroococcus*, *Coelosphaerium*, *Cyanobium*, *Cyanodictyon*, *Merismopedia*, *Romeria*, *Snowella* and *Tetracercus* [10].

Picocyanobacteria of the *Synechococcus* group span a range of different colours, depending on their pigment composition [38,39]. *Synechococcus* sp. consists of strains rich in the pigment phycoerythrin (PE), rendering its representatives a variety of orange, brown, reddish, pink and purple colours and strains rich in phycocyanin (PC), colouring the organism in various shades of blue-green [40]. PE-rich strains of picocyanobacteria are dominant components in open ocean waters, where green and particularly blue light penetrate deeply into the water column. Moreover, red picocyanobacteria can have two different bilin pigments known as phycoerythrobilin (PEB) and phycourobilin (PUB), which both bind to the apoprotein phycoerythrin. PE-rich strains containing relatively high contents of the PUB occur in the clearest ocean waters in which blue light prevails whereas strains rich in PEB occur in more mesotrophic marine waters characterized by blue-green light environments [38,41]. Conversely, PC-rich strains of picocyanobacteria dominate in turbid inland waters in which orange and red light prevail [38,42]. On the other hand, coexistence of PE- and PC-rich picocyanobacteria can be found in waters of intermediate turbidity, such as many freshwater lakes and coastal seas including Baltic Sea [2,38,39,42]. 

**Figure 1:**
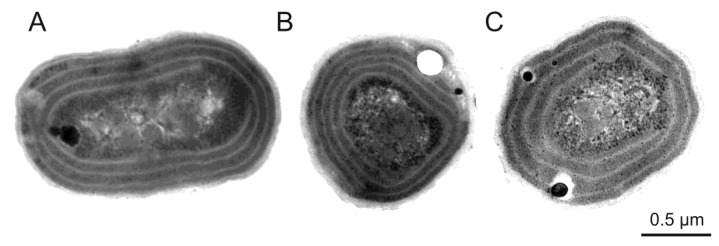
Ultrastructure of three Baltic *Synechococcus* sp. strains: rich in PE (**A**); rich in PC (**B**) and rich in PE containing high contents of the PEB (**C**) analysed using an electron microscope. Photographs by Śliwińska-Wilczewska.

Because of the very small size, there are still great difficulties with the identification of picoplanktonic organisms and the number of well-described taxa is still small. Originally, the classification was based primarily on physiological observations [43]. However, recently used modern techniques, such as epifluorescence microscopy, electron microscopy, flow cytometry and other methods of molecular biology have significantly broadened the criteria for their classification [22,40,44] (Figure 1).

**Figure 2:**
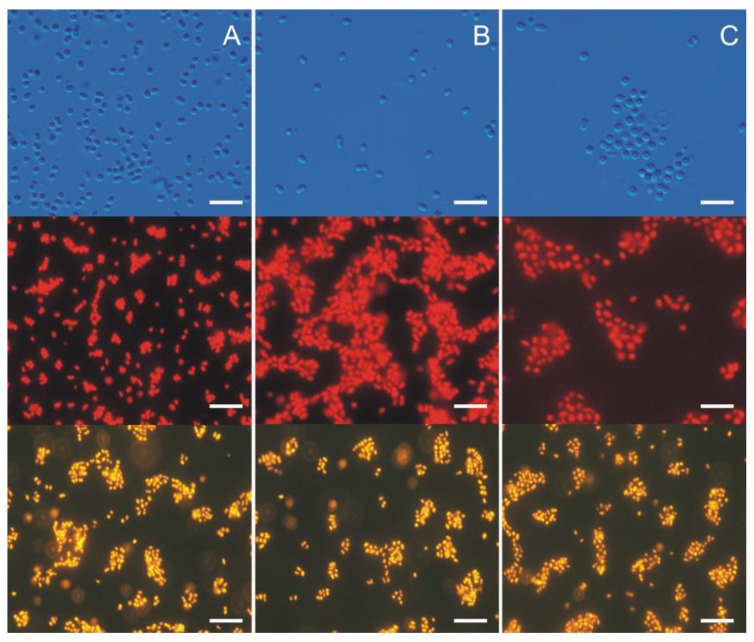
Three Baltic *Synechococcus* sp. strains: rich in PE (**A**); rich in PC (**B**) and rich in PE containing high contents of the PEB (**C**) under a light and epifluorescence microscope. **Top** panel depicts picocyanobacterial cells from light microscope, whilst middle and **bottom** panel illustrates target species under epifluorescence microscope using B-2A and G-2A (which excitation are: 450–490 nm and 510–560 nm, respectively) block filters, respectively. Bar denotes 10 μm. Photographs by Śliwińska-Wilczewska.

Unlike heterotrophic bacteria, picocyanobacteria, due to the presence of chlorophyll *a*, have the capacity for red autofluorescence. Picocyanobacteria are also well distinguishable from picoeukaryote green algae and diatoms because, when used with special filters, their fluorescence is yellow-orange (Figure 2). Therefore, research in fluorescence microscopy enable the initial distinction of eukaryotic and prokaryotic organisms, although the precise taxonomic analysis of picoplanktonic cyanobacteria requires a different approach.

**Figure 3:**
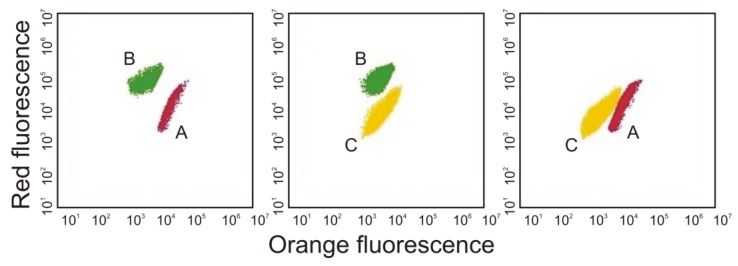
Cytograms obtained with co-cultures with three Baltic *Synechococcus* sp. strains: rich in PE (A), rich in PC (B) and rich in PE containing high contents of the PEB (C)analysed using a Becton Dickinson (BD Biosciences) Accuri™ C6 Plus flow cytometer. Cytograms by Śliwińska-Wilczewska.

Paz-Yepes et al. [45] also noted that it is not possible to distinguish picocyanobacterial strains from one another by light or epifluorescence microscopy techniques. Therefore, they characterized different *Synechococcus* sp. strains in co-cultures using the relative abundance of the *rpoC1* marker gene and specific primers and conditions for each strain. However, it is also possible to discriminated different picocyanobacterial strains using a flow cytometer on the basis of their pigment fluorescence [38,46] (Figure 3). Cells rich in PE emitted orange light (550–620 nm) when excited by the green laser, whereas cells rich in PC emitted far red light (>670 nm) when excited by the red laser [38]. Moreover, the flow cytometer distinguished between picocyanobacteria and larger phytoplankton by their size (using side scattering).

## 4. Blooms of Picocyanobacteria

Over the past few decades, the world’s coastal waters have experienced an increase in the number of harmful algal bloom events. Anderson et al. [47] described that blooms are occurring in more areas than ever before and new massive blooms are reported regularly. Thus, the issues of picocyanobacterial blooms require more attention and interest from researchers.

**Figure 4:**
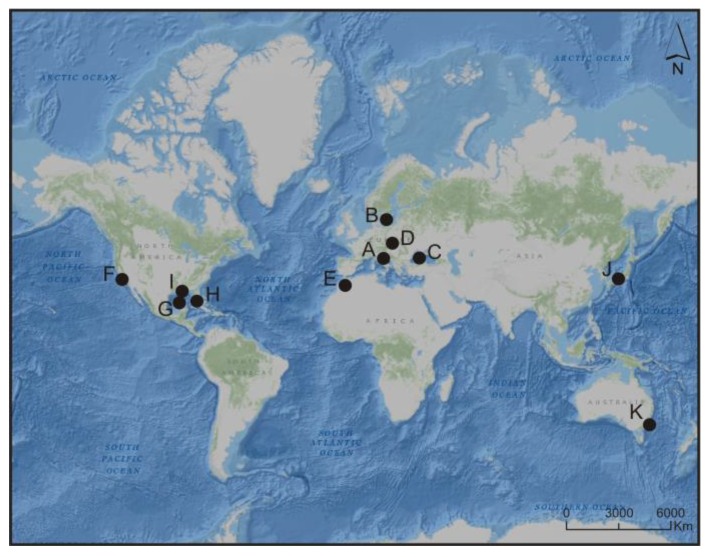
Water reservoirs in which mass occurrence of picoplanktonic cyanobacteria was recorded: Mediterranean Sea (A), Baltic Sea (B), Black Sea (C), Hungarian lakes (D), ponds of Morocco (E), San Francisco Bay (F), Gulf of Mexico (G), Florida Bay (H), Pensacola Bay (I), Seto Inland Sea (J) and Gippsland Lakes (K).

Picocyanobacteria are often described as a non-blooming group (e.g., [48]). However, it is becoming increasingly evident that picocyanobacteria are significant contributors to massive blooms in tropical and subtropical coastal areas and even appear in temperate waters. Significant blooms of picocyanobacteria have been recorded in the Mediterranean Sea [13,15,49], the Baltic Sea [50], the Black Sea [51], Hungarian lakes [52], ponds of Morocco [53,54], San Francisco Bay [55], Gulf of Mexico [56], Florida Bay and Pensacola Bay [30,57,58], the Seto Inland Sea [59] and Gippsland Lakes (Australia) [18] (Figure 4).

The most sufficiently analysed and described bloom of picocyanobacteria occurred in the northern part of the Mediterranean Sea [13,15,49]. In a few research papers, authors described local ecosystem disturbances caused by the mass appearance of picoplankton. Sorokin and Zakuskina [49] showed that the Adriatic coast has been experiencing a super-dense and long-term bloom of picocyanobacteria. The density of the picocyanobacterial bloom varied from 8 to 35 × 10^6^ cells mL^−1^ and picocyanobacterial fraction of the total phytoplankton biomass was 98% from spring till early summer and 92% in autumn. The eutrophication phenomenon in the coastal ecosystem of Comacchio (Mediterranean Sea) for the first time resulted in the bloom of picocyanobacteria in 1985. During this period, an extremely dense biomass of picoplanktonic cyanobacteria and its negative effects on the environment was a new, unprecedented phenomenon in Europe. Persistent blooming of picocyanobacteria resulted in typical hypereutrophication effects such as: drastic increase in turbidity resulting in death of the benthic flora due to light deficiency; accumulation of organic matter and total phosphorus in the water column and in the sediment; anaerobic conditions in the bottom layer as well as sulphide accumulation in the sediment. Previously, the bottom of the lagoon was covered with numerous species of macrophytes from the genera *Valonia*, *Lamprothamnium*, *Chaetomorpha* and *Ruppia*. During blooms, benthic vegetation almost completely disappeared and was replaced by microbial mats. The negative impact on the ecosystem has been also enhanced by the toxic effects on animals, which are important links in the pelagic trophic chain. The bloom was accompanied by drastic changes in the benthic communities and the share of filtering fauna in the whole ecosystem metabolism was less than 2%. Moreover, the bloom in the Comacchio lagoons was formed mainly by picocyanobacteria loosely suspended in the mucus, which made the filter clams and the copepods unable to feed on them. The colonies of cyanobacteria protect themselves against their own ingestion [60]. Furthermore, *Cyanocystis* sp. and *Coelosphaerium* sp., observed in the Comacchio lagoons, belong to toxic organisms [61]. Therefore, the significant reduction in the number of organisms that typically feed on picoplankton that was observed might be caused by their toxins. However, the toxicity of the bloom-forming picocyanobacteria from the Comacchio lagoons is unknown and this is a significant issue with regard to management and public health problems. In addition, these extremely dense blooms of picoplanktonic cyanobacteria dominated the Comacchio ecosystem for many years without showing any seasonal or long-term changes [49]. Furthermore, Sorokin et al. [13] and Sorokin and Dallocchio [15] recorded picocyanobacterial bloom (5–20 × 10^6^ cells mL^−1^) in the Venice lagoon (Mediterranean Sea) in the summer period. Sorokin et al. [13] examined that the share of picocyanobacteria of the total phytoplankton varied in the Venice lagoon in July–September from 60 to 98%. Authors noted that the populations of micro- and mesozooplankton were found to be inhibited in areas of intensive picocyanobacterial bloom. Additionally, a significant mortality of key species for the local fishery was recorded. In this study authors clearly demonstrated the harmful effect of the bloom of picoplanktonic organisms that has appeared in several Adriatic lagoons. It was also suggested that the blooms of picocyanobacteria in lagoons appear to be far more disastrous for their resources and environmental state than even toxic dinoflagellate blooms. Moreover, the bloom displays contagious features and shows a tendency to become persistent.

Previous investigations also confirmed that in the temperate zone, picocyanobacterial blooms were observed in summer while picoeukaryotes blooms occurred in winter. Kuosa [50] noted that picoeukaryotes in the Baltic Sea were abundant during the whole year (10^3^–10^4^ cells mL^−1^), while picocyanobacterial blooms occurred only during summer period (10^5^–10^6^ cells mL^−1^). In the Black Sea, surface blooms of *Synechococcus* (10^5^ cells mL^−1^) were also reported [51]. Water temperatures of 5–10 °C, resulted in large winter picoplankton blooms also in the Büdös-szék (108 × 10^6^ cells mL^−1^), Kelemen-szék (50 × 10^6^ cells mL^−1^) and Zab-szék (47 × 10^6^ cells mL^−1^) lakes (Hungary) [52]. The authors explained that the low temperature and low light intensity in winter provide a competitive advantage to picoeukaryotes, while higher temperatures and higher light intensity are more favourable for picocyanobacteria.

Furthermore, picoplanktonic organisms are able to create harmful blooms in tropical and sub-tropical waters. The occurrence of a summer *Synechocystis* sp. bloom in the wastewater treatment plants of Marrakech was studied by Mezrioui and Oudra [53] and Oudra et al. [54]. It was suggested that the organisms responsible for this phenomenon could be transferred by migratory birds from North Africa. A similar phenomenon has been observed in marine ecosystems. Ning et al. [55] showed that picocyanobacterium *Synechococcus* sp. was a persistent component of phytoplankton in all the estuarine habitats in the San Francisco Bay with peak abundance during the spring bloom (5.2 × 10^5^ cells mL^−1^). This result indicated increasing importance of picocyanobacteria along the gradient of decreasing nutrient concentrations from estuaries to the open ocean. In addition, Wawrik and Paul [56] have clearly demonstrated the importance of nutrient inputs from the Mississippi River in stimulating *Synechococcus* blooms in the Gulf of Mexico. The massive blooms of *Synechococcus* sp. (10 × 10^6^ cells mL^−1^) were also observed by Philips et al. [57] and Gardner and McCarthy [58] in the north-central region of Florida Bay. Murrell and Lores [30] noted that the *Synechococcus* sp. bloom occurred in the Pensacola Bay during summer, exceeding 3 × 10^6^ cells mL^−1^, and was strongly correlated with temperature. Moreover, the authors noted that the increase of copepods *Oithona* sp. coincided with an increase of picophytoplankton. Similarly, Nakamura et al. [59] noted that following the picoplankton bloom (3–6 × 10^5^ cells mL^−1^), the abundance and biomass of appendicularia *Oikupleuradioica* in the Seto Inland Sea increased rapidly by ingesting picoplankton populations. Thus, the trophic implications of picocyanobacterial dominance in tropical and sub-tropical waters need to be more precisely assessed. Moreover, a massive and persistent bloom of picocyanobacterium *Synechococcus* sp., appeared in Gippsland Lakes (Australia) [18]. Author described that it is likely that high temperature and relatively high salinity provide ideal conditions for initiation of the picocyanobacterial bloom.

Many of these observations confirmed the persistence of picocyanobacterial blooms. Sorokin and Zakuskina [49] suggested that the main reason for this phenomenon was the lack of control of the populations of picoplankton organisms by consumers, due to the collapse of filter fauna and the potential mixotrophy of picoplanktonic cyanobacteria. Furthermore, the dominance of picocyanobacteria may be attributable to several of the unique physicochemical characteristics of these organisms, including its small size, cyanobacterial metabolism, euryhaline character, buoyancy and tolerance to high light intensity [57]. It is also possible that picocyanobacteria are achieving a competitive advantage over other organisms due to their wide potential to adapt to changing environmental conditions (e.g., [3,18,22]) and their allelopathic activity (e.g., [28,29,62,63]). The results of previous study confirmed the dangerous character of the picocyanobacterial bloom in aquatic environment. Moreover, described blooms tend to spread and remain for a long time and cause drastic, adverse changes in aquatic ecosystems. Therefore, more efforts have to be done to investigate in depth the autecology and taxonomy of bloom-forming picocyanobacteria and their effect on surrounding ecosystems.

## 5. Picocyanobacterial Secondary Metabolites

Picocyanobacteria have been rarely studied with respect to their potential as producers of bioactive compounds because most of previous works have assumed a lack of toxin production by these cyanobacteria. However, picoplanktonic cyanobacteria belong to the organisms that produce wide range of secondary metabolites (Table 1). These organisms are also capable of secreting compounds that may be bioaccumulated or biomagnified (e.g., [64,65]). Consequently, their harmful effects on the environment may be related to further links in the trophic chain and also affect humans [66,67,68]. 

The microcystins (MCs) are cyclic heptapeptides and are by far the most studied of the cyanobacterial toxins [67]. The toxicity of picoplanktonic cyanobacteria *Synechocystis* sp. and *Synechococcus* sp. was described for the first time by Lincoln and Carmichael [69] and Mitsui et al. [70]. Almost a decade later, picocyanobacterial toxicity was also confirmed by Domingos et al. [71] and Bláha and Maršálek [72]. Domingos et al. [71] noted that six strains of picocyanobacteria from northern Brazil are capable of producing toxic MCs. Four of these strains have been identified as colonial *Aphanocapsa cumulus*. The other two strains formed loosely distributed cells making their classification impossible. Domingos et al. [71] examined that concentrations of picocyanobacterial MCs were very low ranging from 0.08 to 3.7 µg g^−1^ dry weight. Additionally, HPLC analysis of three picocyanobacteria—*Synechococcus nidulans*, *Cyanobium rubescens* and *Cyanobacterium cedrorum*—indicated that they produce MCs [72]. Later studies have also confirmed the release of MC-LR and MC-YR (variants in which the two variable amino acids are leucine-L and argenine-R and tyrosine-Y and argenine-R, respectively) by the *Synechococcus* sp. strain (SS-1) isolated from the Salton Sea, the largest inland body of water in California [73]. Other studies also have shown that two strains of *Synechococcus* sp. (63a-1 and 63a-3) isolated from Florida Keys (Atlantic Ocean) are capable of producing small amounts of MC-LR (0.27 μg g^−1^ and 0.08 μg g^−1^, respectively) [74]. In turn, Martins et al. [75] screened the picocyanobacterial strains for microcystins by ELISA and examined that *Synechococcus* sp. and *Synechocystis* sp. strains produced MCs in small quantities. Subsequent results showed that ELISA tests specific for hepatotoxic MCs gave positive results for two strains of picocyanobacteria, *Synechococcus* sp. (CENA108) and *Merismopedia* sp. (CENA106) [76]. Detection of MCs from two *Synechocystis* sp. strains (Syn-WTP93 and Syn-WTP97) isolated from freshwater reservoirs in Morocco was also described by Oudra et al. [77]. The concentrations of MCs for these strains determined by ELISA varied from 15 to 56 μg g^−1^ dry weights, respectively. Vareli et al. [78] indicated that picocyanobacteria *Synechococcus* sp. and *Synechocystis* sp. were responsible for the presence of MCs in the Amvrakikos Gulf (Mediterranean Sea). Authors noted that the cyanobacterial community was found to be dominated almost exclusively by the picoplanktonic cyanobacteria *Synechococcus* sp. and *Synechocystis* sp. Marine *Synechococcus* sp. and *Synechocystis* sp. accounted for more than 50% of the total cyanobacterial biomass in the World Ocean. Thus, picocyanobacterial species should be considered also as significant MCs producers [65]. In the initial phase of blooms, hepatotoxins (MCs and nodularin, NOD) occur inside the cyanobacteria cells however, after cell lysis, the release of toxic metabolites causes the increase of their concentration in the water [79]. Therefore, Carmichael and Li [73] and Vareli et al. [65] suggested that the production of MCs by picoplanktonic cyanobacteria indicates that these toxins may be a more common occurrence in aquatic environments than previously thought. On the other hand, the secretion of NOD by picoplanktonic organisms remains still unconfirmed and the research on this subject is insufficient [10,79]. In spite of all, the possibility of producing toxins by picoplanktonic cyanobacteria is especially relevant if we take into consideration that these organisms are one of the most ubiquitous components in freshwater, brackish and marine ecosystems. 

It was also demonstrated that the neurotoxic nonprotein amino acid, *β*-*N*-methylamino-l-alanine (BMAA), may be produced by all known groups of cyanobacteria [80]. Literature reports have demonstrated the synthesis and secretion of BMAA by the freshwater strain of *Synechococcus* sp. (PCC 6301) and the marine strain of *Prochlorococcus marinus* (CCMP1377). The authors examined that the content of free BMAA for *Synechococcus* sp. and *P. marinus* was 25 μg g^−1^ and 32 μg g^−1^, respectively [80]. Cianca et al. [81] also reported the detection of BMAA in five picocyanobacterial strains of *Cyanobium* sp. (LEGE 06068), *Synechocystis salina* (LEGE 06079), *Synechocystis* cf. *salina* (LEGE 06083), *S*. cf. *salina* (LEGE 07073) and *Synechococcus* sp. (LEGE 07074), isolated from the Portuguese estuaries of Minho, Douro and Vouga Rivers. It was demonstrated that the content of picocyanobacterial BMAA depended on the picocyanobacterial strain and extraction technique (Methanol/Acetone extraction, followed by HCl extraction, TCA extraction and HCl extraction). Authors examined that the values obtained varied from 0.04 μg g^−1^ for *Cyanobium* sp. sequential extraction with Methanol/Acetone plus HCl to 63 μg g^−1^ for *S. salina* extraction with HCl. Cox et al. [82] suggested that alternative ecological pathways for the bioaccumulation of cyanobacterial BMAA in aquatic or terrestrial ecosystems may cause increasing concentrations of toxic compounds up the food chain. Cyanobacterial BMAA has been also associated with certain forms of progressive neurodegenerative human diseases [81]. Therefore, Cox et al. [80] suggested that because of the global importance of picocyanobacterial blooms, a broader analysis of the production of BMAA in aquatic ecosystems is strongly needed.

Recent investigations suggest that picocyanobacterium *Synechococcus* sp. synthesize 2-methylisoborneol (MIB) and geosmin (1,2,7,7-tetramethyl-2-norbomeol) (GSM) [79,83,84]. It was also demonstrated that MIB is produced and secreted during cyanobacterial cell cycle, while GSM is released only after cells’ death. The toxic effects of these compounds to other organisms have not yet been demonstrated but they influence the quality of water and organisms living there [10]. Moreover, it has been suggested that picocyanobacteria might not only produce these compounds but also take part in their transfer to higher trophic levels [84].

For many organisms, lipopolysaccharides (LPS) are the first defence against unfavourable factors [85]. Schmidt et al. [86,87], examined that picocyanobacteria *Synechococcus* sp. and *Synechocystis* sp. contained LPS in their cell wall. Authors found that the LPS of eight strains of *Synechococcus* sp. as well as four strains of *Synechocystis* sp. contained fucose, mannose, galactose, glucose and glucosamine. Additionally, LPS obtained from *Synechococcus* sp. exhibited little reactivity in antisera raised in rabbits against homologous cells while the LPS from *Synechocystis* sp. showed no specific activity. Moreover, Snyder et al. [85] demonstrated that two strains of marine *Synechococcus* sp. (WH8102 and CC9311) had a very simplified structure of LPS which showed lack of limulus amoebocyte lysate gelation activity. Authors suggested that the highly simplified nature of picocyanobacterial LPS may cause their adaptation to the relatively higher salt levels in marine environments. On the other hand, liposacharids have strongly allergic and irritating effects and also cause decreased activity of glutathione S-transferases, which participate in the detoxification of xenobiotics [88]. However, a harmful or allelopathic effect of picocyanobacterial LPS has not yet been investigated.

Among unique picocyanobacterial bioactive compounds that may influence various organisms are a thioic *O*-acid ester-containing sulfolipid (thionsulfolipid). Thionsulfolipid was isolated from cells of picocyanobacterium *Synechococcus* sp. [89]. The authors clearly demonstrated that this thionsulfolipid was toxic to fish (*Tanichthys albonubes*) and caused growth inhibition of human lymphoma cells at a concentration of 200 μg mL^−1^. Kunimitsu et al. [89] noted that picocyanobacterial thionsulfolipid has not been found in any other photosynthetic organisms. Additionally, Liu et al. [90] discovered a new stereoisomer of an araguspongine/xestospongin alkaloid—named araguspongine M—which has been isolated from the marine sponge *Neopetrosia exigua* (formerly *Xestospongia exigua*), collected in Palau. Authors noted that this compound may be produced by an endosymbiotic *Synechococcus*-like cyanobacterium. Moreover, structurally related to schizokinen, a citrate-derived hydroxamate siderophore, the synechobactins A–C were isolated from the marine cyanobacterium *Synechococcus* sp. strain (PCC 7002), grown under iron-limiting conditions [91]. Synechobactins A–C differ among themselves in the identity of the fatty acid residue as dodecanoic acid, decanoic acid, or octanoic acid, respectively. Another approach is genetic modification of *Synechocystis* sp. strain PCC6803 to produce and secrete fatty acids [92]. However, no harmful effect of these picocyanobacterial compounds has been examined. Therefore, further research should be done to define the distribution of secondary metabolites produced and released by picoplanktonic cyanobacteria and to determine the possible harmful effects of these compounds on other organisms.

## 6. Allelopathic Activity of Picocyanobacteria and Their Impact on Aquatic Environment

Allelopathy is considered as one of the factors promoting and maintaining the massive cyanobacterial and algal blooms in freshwater, brackish and marine ecosystems around the world [93,94,95,96]. Therefore, the number of reports about the allelopathic effects of different species of cyanobacteria and microalgae has been steadily increasing (e.g., [95,96,97,98,99]). However, only little information on picocyanobacterial allelopathy or their harmful effects on other organisms has been described (Table 2). 

The first observation of allelopathic interactions between picocyanobacteria *Synechococcus* sp. was noted by Paz-Yepes et al. [45]. In this work authors used a liquid and plate assays to determine whether these interactions occur between mentioned marine picocyanobacterial strains: CC9311, WH8102 and CC9605. It was found that *Synechococcus* sp. CC9605 always dominated when co-cultured with CC9311 or WH8102 in liquid medium. These effects were also seen in solid medium. When a spot of CC9605 was plated on an existing lawn of CC9311 or WH8102, a zone of clearing developed, which was 5-fold larger in the case of WH8102. Surprisingly, no evidences were found of allelopathic effects of these two strains against *Synechococcus* sp. CC9605. Additionally, *Synechococcus* sp. CC9311 dominated the co-culture when growing with *Synechococcus* sp. WH8102, while no reciprocal effect was observed. Moreover, Paz-Yepes et al. [45] suggested that a Microcin C-like molecule is involved in the allelopathic interactions with *Synechococcus* strain CC9605. On the other hand, it was found that *Synechococcus* sp. CC9311, which does not encode an McC-like gene cluster, noticeably inhibits the growth of *Synechococcus* sp. WH8102, presumably by the production of a different allelochemical. 

On the other hand, Śliwińska-Wilczewska et al. [28] described for the first time that the Baltic strain of *Synechococcus* sp. (CCBA-124)affects coexisting diatom *N. perminuta* negatively. These studies indicated that high light and temperature and low salinity affected the tested picocyanobacteria by increasing its allelopathic activity. Authors noted that the highest decline in diatom growth, chlorophyll *a* fluorescence and photosynthesis was observed after the addition of cell-free filtrate obtained from culturesgrown at 190 μmol photons m^−2^ s^−1^, 25 °C and 8 PSU which coincided with optimal *Synechococcus* sp. growth conditions. Moreover, these results demonstrated that variation in light intensity, water temperature and salinity should be considered when estimating the potential effects of picocyanobacterial allelopathy in aquatic environments. Additionally, Śliwińska-Wilczewska et al. [63] demonstrated that the picocyanobacterium *Synechococcus* sp. (CCBA-124) is capable of secreting unidentified allelopathic compounds that have a negative impact on the whole phytoplankton assemblages. Authors examined the influence of allelopathic compounds on the growth, total abundance and structure of phytoplankton community by single and multiple addition of cell-free filtrate of picocyanobacterium *Synechococcus* sp. In this work, it was demonstrated that the growth of the plankton community was inhibited after exposure to the compounds released by *Synechococcus* sp. After one week of exposure, the chlorophyll *a* and chlorophyll *c* concentration was lower in the treatment with picocyanobacterial filtrate than in the control. Moreover, this study indicated that diatoms of the genera *Navicula*, *Chaetoceros*, *Amphora*, *Coscinodiscus*, *Grammatophora* and *Nitzschia* are the most affected organisms. It was suggested that the allelopathic activity exhibited by the picocyanobacterium *Synechococcus* sp. is probably one of the major competitive strategies affecting some of the coexisting phytoplankton species in aquatic ecosystems.

An interesting concept in the evolutionary context is the allelopathic interaction between coexisting picoplanktonic and filamentous cyanobacteria. Śliwińska-Wilczewska et al. [29,62] showed that Baltic picocyanobacterium of the genus *Synechococcus* are able to produce and release unidentified compounds that have allelopathic effect on the selected filamentous cyanobacteria. In these two papers authors described the negative effects of *Synechococcus* sp. filtrate against *Nodularia spumigena* [29], *Nostoc* sp. and *Phormidium* sp. [62]. It was examined that the negative effects against cyanobacteria were amplified by repeated filtrate additions compared with single filtrate addition. Moreover, the authors showed that the addition of picocyanobacterial filtrate stimulated the growth of *Aphanizomenon flos-aquae* and had no allelopathic effects on *Rivularia* sp. [62]. These results demonstrated for the first time that picocyanobacterium *Synechococcus* sp. negatively and positively affected coexisting filamentous cyanobacteria however, the identification of the allelopathic compounds is necessary to better understand the molecular targets in the affected species.

In the research conducted by Martins et al. [100] aqueous extracts and organic solvent extracts of isolated marine picocyanobacteria strains *Synechococcus* sp. and *Synechocystis* sp. were tested for antimicrobial activity against a fungus, Gram-positive and Gram-negative bacteria. In addition, cytotoxic assays have been also performed using primary rat hepatocytes and HL-60 human monocytic leukaemia cells. The work showed that the picocyanobacterial strains were found to have antibiotic activity against two Gram-positive bacteria, *Clavibacter michiganensis* subsp. *insidiosum* and *Cellulomonas uda*, while no inhibitory effects were found against the fungus *Candida albicans* and Gram-negative bacteria. The results obtained in this study also showed intense effect of the extracts on monocytes and a slight apoptotic effect in the case of hepatocytes, which may indicate that the tested cyanobacteria produce unrecognized toxic substances in small amounts, or the effect of these substances is not very strong. The authors have suggested that the effects on hepatocytes may be delayed and changes in these cells may intensify only after prolonged exposure. In turn, Costa et al. [101] assessed the anticancer potential of extracts from fifteen marine picocyanobacteria strains, belonging to the picoplanktonic genera, *Cyanobium*, *Synechocystis* and *Synechococcus*. In this work, picocyanobacteria crude extract and fractions obtained by chromatography were tested in eight cancer cell lines, which were selected as being representative of several human tumours. Authors found that eight strains of *Cyanobium* sp., one strain of *Synechococcus nidulans*, three strains of *Synechococcus* sp. and three strains of *Synechocystis salina* were able to induce cytotoxic effects in human cancer cell lines. It was noted that the remaining 59% of strains, considered as having no cytotoxic effect however, the majority of the tested picocyanobacterial strains were capable of inducing cytotoxicity in at least one of the cell lines. Moreover, strain *Synechocystis salina* LEGE 06155 was one of the most cytotoxic strains with above the 90th percentile of the standardized effect on cancer cell viability, classified as a strong effect. Furthermore, Liu et al. [90] described that araguspongine M, produced by an endosymbiotic *Synechococcus*-like cyanobacterium, showed cytotoxicity against the human leukaemia cell line HL-60 but did not inhibit the growth of bacteria *Escherichia coli*, *Staphylococcus aureus*, *Saccharomyces cerevisiae*, *Mucor hiemalis* and *Ruegeria atlantica*. On the other hand, Noaman et al. [102] showed that an antimicrobial agent produced by the cyanobacterium *Synechococcus leopoliensis* was active against the Gram-positive bacterium *Staphylococcus aureus*. Authors examined that temperature 35 °C, pH 8 and 15 days of incubation in the medium BG-11 were the best for antimicrobial agent production. Leucine was the best nitrogen source for antimicrobial activity. It was also found that glucose at a concentration of 0.6 mg L^−1^ was the most optimal, while mannitol and xylose were not suitable carbon sources for antimicrobial agent production of *S. leopoliensis*. Authors suggested that the high levels of antimicrobial activity of *S. leopoliensis* could be attributed to the chemical nature of the supplements.

In another work, Martins et al. [103] described the effects of picocyanobacterial strains *Synechococcus* sp. and *Synechocystis* sp. isolated from the marine coast of Portugal on selected marine invertebrates. In this work, crude and partially purified extracts of the picocyanobacterial strains were tested for acute toxicity in nauplii of the brine shrimp *Artemia salina*, in the rotifer *Brachionus plicatillis* and in embryos of the sea urchin *Paracentrotus lividus* and the mussel *Mytilus galloprovincialis*. According to the results obtained in the assay using *A. salina*, both *Synechococcus* sp. and *Synechocystis* sp. strains displayed a negative effect on the survival of the nauplii, while the crude extracts of selected strains revealed an evident toxic effect by causing 100% mortality after 24 h of exposure. On the other hand, no significant toxic effects were registered against the rotifer *B. plicatillis*. Moreover, the toxic effect of the different extracts of the picocyanobacterial strains was evident for the embryos of the sea urchin *P. lividus* and the mussel *M. galloprovincialis*, with toxic effects resulting in an inhibition of embryogenesis or development of smaller larvae. To the mussel embryos, the effects of picocyanobacterial extracts resulted in a complete inhibition of embryogenesis. The results revealed the ability of marine *Synechococcus* sp. and *Synechocystis* sp. crude and partially purified extracts to be toxigenic to early life stages of marine invertebrates. However, the compounds responsible for this action have not been identified [103]. Other studies have also shown that picocyanobacteria *Cyanobium* sp. (LEGE 06008, 06011 and 06015) and *Synechococcus* sp. (LEGE 06005) isolated from rocky beaches along the Atlantic Portuguese central coast caused acute toxicity in nauplii of the brine shrimp *Artemia salina* [104]. In this work, results concerning the methanolic and the aqueous extract were more pronounced for most of the picocyanobacterial strains, with *Synechococcus* sp. LEGE 06005 and *Cyanobium* sp. LEGE 06015 reaching 100% nauplii mortality with the aqueous extract. Authors emphasized that the strongest toxic effect was noted with aqueous solutions, suggesting that the toxins produced by the studied organisms dissolve in water [104]. Moreover, Costa et al. [105] examined the toxicological potential of five marine picocyanobacterial *Cyanobium* sp. strains (LEGE 06098, 06134, 06139, 07175, 07186) isolated from the Portuguese coast using different biological models. A crude extract and three fractions reflecting a preliminary segregation of lipophilic metabolites were tested for picocyanobacterial toxicity with the microalga *Nannochloropsis* sp., the bacteria *Pseudomonas* sp., the brine shrimp *Artemia salina* and fertilized eggs of the sea urchin *Paracentrotus lividus*. Authors described that *Cyanobium* sp. strains inhibited *Nannochloropsis* sp. and *Pseudomonas* sp. growth and induced a decrease in *P. lividus* larvae length. On the other hand, no significant apparent adverse effects were noted against *Artemia salina*. The results obtained indicated that *Cyanobium* genus may serve as a potential source of interesting bioactive compounds and emphasize the importance of studying the smallest fractions of marine cyanobacteria.

Recent work suggests that picocyanobacteria of the genus *Synechococcus* may also contribute to behavioural changes and locomotor disorders in vertebrates. In a paper published by Hamilton et al. [106], the effect of two strains of *Synechococcus* sp. (CC9311 and CC9902) on the fish of the species *Embiotoca jacksoni* was analysed. Laboratory experiments have shown that organisms exposed to picocyanobacteria are characterized by reduced mobility, moving much slower and spending more time motionless. In addition, the examined fish preferred dark zones of experimental tanks, which may be caused by stress and result from seeking refuge from the negative impact of *Synechococcus* sp. It was suggested that such changes may be the result of secondary metabolites that are absorbed through the gills and then cross the blood-brain barrier, disturbing the functioning of the neurological system. The experiments also showed that the return to clean water tanks resulted in gradual disappearance of negative effects of exposure to the direct contact with picocyanobacterium *Synechococcus* sp. This may mean that the compounds produced by *Synechococcus* sp. reduce the condition of vertebrates and cause behavioural disorders but do not lead to permanent changes in organisms. As a result, it can be assumed that the large quantities of *Synechococcus* sp. may be associated with negative, although relatively brief, transient effects on the aquatic environment. It was also found that picocyanobacterial strains of the genus *Cyanobacterium*, *Synechococcus* and *Synechocystis* isolated from rocky beaches along the Portuguese coast are able to produce unrecognized, toxic substances, as confirmed by studies in mice [75]. Authors demonstrated that the toxicity to mice was observed after injection of centrifuged and non-centrifuged picocyanobacterial extracts. Experiments reported a reduced rate of respiration and imbalance in those animals affected by the picocyanobacterial compounds. Furthermore, neurotoxic symptoms and effects in liver, kidney, small intestine and lungs was also examined [75]. All the results obtained indicated that the studied picocyanobacteria are a promising source of novel compounds with allelopathic activity.

Despite the ecological importance of picoplanktonic cyanobacteria, very little is known about their allelopathic effects on other organisms. Picocyanobacteria are known to produce a variety of bioactive compounds, whose harmfulness to animals and humans has been clearly demonstrated. At the same time the functional role of these compounds, particularly in terms of the physiology and ecology of the cyanobacteria that produce them, remains largely unknown. Production and release of allelopathic compounds with different properties can give producing species a competitive advantage and build their effective strategy. Furthermore, secreted allelopathic compounds by picocyanobacteria may be responsible for the natural selection of organisms and ecological succession. The results obtained indicated that picocyanobacteria may serve as a potential source of interesting bioactive compounds, which mode of action on target organisms required detailed investigation. Furthermore, the increasing occurrence of picocyanobacteria in large densities at aquatic habitats resulting from eutrophication and global warming poses a serious threat to humans and ecosystems. Clearly there is a need to increase our knowledge about allelochemicals production by picocyanobacteria strains, in order to prevent possible adverse effects of its occurrence.

## 7. Conclusions

Picocyanobacteria are common in freshwater, brackish and marine ecosystems throughout the world and play an important role in the functioning of aquatic ecosystems. However, despite their ubiquity, picocyanobacteria are still a group of poorly understood organisms. So far, only a few reports have been published discussing the secretion of toxins and other allelopathic compounds as well as creating harmful blooms by picoplanktonic cyanobacteria. Cyanobacterial blooms are a major and growing problem for freshwater and marine ecosystems worldwide that increasingly concerns public health. These toxins are collectively responsible for human fatalities, as well as continued and widespread poisoning of wild and domestic animals. Picocyanobacterial blooms may also cause the death of a large part of both fauna and benthic flora, as well as economic losses. In addition, some picocyanobacteria were capable of secondary metabolites production which may also be a source of harmful bioactive compounds. A dense bloom of picocyanobacteria is a new phenomenon, which still needs further investigation. While the interactive effects of climate change on harmful picocyanobacterial blooms are complex, much of the current knowledge suggests that these processes are likely to enhance the magnitude and frequency of these events. Therefore, it is essential to carefully examine the role of these small organisms in aquatic ecosystems.

## Figures and Tables

**Table 1:** Secondary metabolites produced by picocyanobacteria and their source of origin.

Species (Strain)	Location/Habitat	Secondary Metabolites	Source
*Aphanocapsa cumulus*	Caruaru reservoirs (Brazil)/freshwater	MC	[71]
*Synechococcus nidulans*	Unknown/freshwater	MC	[72]
*Cyanobium rubescens*	Unknown/freshwater	MC	[72]
*Cyanobacterium cedrorum*	Unknown/freshwater	MC	[72]
*Synechococcus* sp. (SS-1)	Salton Sea (California)/marine	MC	[73]
*Synechococcus* sp. (63a-1 and 63a-3)	Florida Keys (Atlantic Ocean)/marine	MC	[74]
*Synechococcus* sp.	Portuguese coast (Atlantic Ocean)/marine	MC	[75]
*Synechocystis* sp.	Portuguese coast (Atlantic Ocean)/marine	MC	[75]
*Synechococcus* sp. (CENA108)	Cajati (Brazil)/freshwater	MC	[76]
*Merismopedia* sp. (CENA106)	Cajati (Brazil)/freshwater	MC	[76]
*Synechocystis* sp. (Syn-WTP93 and Syn-WTP97)	Biological wastewater treatment plant (Morocco)/freshwater	MC	[77]
*Synechococcus* sp.	Amvrakikos Gulf (Mediterranean Sea)/marine	MC	[78]
*Synechocystis* sp.	Amvrakikos Gulf (Mediterranean Sea)/marine	MC	[78]
*Synechococcus* sp. (PCC 6301)	USA/freshwater	BMAA	[80]
*Prochlorococcus marinus* (CCMP1377)	Sargasso Sea (Atlantic Ocean)/marine	BMAA	[80]
*Cyanobium* sp. (LEGE 06068)	Douro estuary/brackish	BMAA	[81]
*Synechocystis salina* (LEGE 06079)	Douro estuary/brackish	BMAA	[81]
*Synechocystis* cf. *salina* (LEGE 06083)	Douro estuary/brackish	BMAA	[81]
*Synechocystis* cf. *salina* (LEGE 07073)	Vouga estuary/brackish	BMAA	[81]
*Synechococcus* sp. (LEGE 07074)	Douro estuary/brackish	BMAA	[81]
*Synechococcus* sp.	Lake Bowen and Municipal Reservoir #1 (USA)/freshwater	MIB	[83,84]
*Synechococcus* sp.	Lake Bowen and Municipal Reservoir #1 (USA)/freshwater	GSM	[83,84]
*Synechococcus* sp. (PCC 6907, 6307, 6911, 6603, 6908, 6311, 6312, 6910)	France/freshwater	LPS	[86]
*Synechocystis* sp. (PCC 6714, 6803, 6807, 6308)	France/freshwater	LPS	[87]
*Synechococcus* sp. (WH8102 and CC9311)	Carribean Sea (Atlantic Ocean) and Pacific Ocean/marine	LPS	[85]
*Synechococcus* sp.	Unknown	thionsulfolipid	[89]
endosymbiotic *Synechococcus*-like cyanobacterium	marine sponge *Neopetrosia exigua*, Palau (Pacific Ocean)/marine	araguspongine M	[90]
*Synechococcus* sp. (PCC 7002)	USA/marine	synechobactins A–C	[91]
*Synechocystis* sp. (PCC6803/SD100)	France/freshwater	fatty acids	[92]

BMAA, *β*-*N*-methylamino-l-alanine. GSM (geosmin), 1,2,7,7-tetramethyl-2-norbomeol. LPS, lipopolysaccharides. MC, microcystin. MIB, 2-methylisoborneol.

**Table 2:** Allelopathic activity of picocyanobacteria and their effect on target organisms. − indicates inhibiting effects, + indicates stimulating effect, 0—indicates lack of effect.

Donor Species (Strain)	Target Species	Effect	Source
*Synechococcus* sp. (CC9311)	*Synechococcus* sp. (WH8102)	−	[45]
*Synechococcus* sp. (WH8102)	*Synechococcus* sp. (CC9311)	0	[45]
*Synechococcus* sp. (CC9605)	*Synechococcus* sp. (CC9311), *Synechococcus* sp. (WH8102)	−	[45]
*Synechococcus* sp. (CC9311), *Synechococcus* sp. (WH8102)	*Synechococcus* sp. (CC9605)	0	[45]
*Synechococcus* sp. (CCBA-124)	*Navicula perminuta*	−	[28]
*Synechococcus* sp. (CCBA-124)	*Nodularia spumigena*	−	[29]
*Synechococcus* sp. (CCBA-124)	*Nostoc* sp., *Phormidium* sp.	−	[62]
*Synechococcus* sp. (CCBA-124)	*Aphanizomenon flos-aquae*	+	[62]
*Synechococcus* sp. (CCBA-124)	*Rivularia* sp.	0	[62]
*Synechococcus* sp. (CCBA-124)	*Navicula* sp., *Chaetoceros* sp., *Amphora* sp., *Coscinodiscus* sp., *Grammatophora* sp., *Nitzschia* sp.	−	[63]
*Synechocystis* sp. (LEANCYA 1, 5, 13, 15, 17, 20, 21) and *Synechococcus* sp. (LEANCYA 7, 10, 11, 16, 18, 19, 22)	*Candida albicans*	0	[100]
*Synechocystis* sp. (LEANCYA 1, 5, 13, 15, 17, 20, 21) and *Synechococcus* sp. (LEANCYA 7, 10, 11, 16, 18, 19, 22)	*Cellulomonas uda*, *Clavibacter michiganensis* subsp. *insidiosum*	−	[100]
*Synechocystis* sp. (LEANCYA 1, 5, 13, 15, 17, 20, 21) and *Synechococcus* sp. (LEANCYA 7, 10, 11, 16, 18, 19, 22)	*Aeromonas hydrophila*, *A. salmonicida* subsp. *salmonicida*, *Bacillus cereus*, *B. megaterium*, *Enterobacter cloacae*, *Escherichia coli*, *Halomonas aquamarina*, *H. pacifica*, *Micrococcus luteus*, *Photobacterium damselae* subsp. *piscicida*, *Proteus vulgaris*, *Pseudomonas doudoroff*, *Staphylococcus epidermidis*, *S. aureus*, *S. parauberis*, *Thiobacillus thioparus*, *Vibrio compbelli*, *V. harveyi*, *V. natriegens*, *V. parahemolyticus*, *V. fluvialis*, *V. tubiashii*, *V. vulnificus*, *Yersinia ruckeri*	0	[100]
*Synechocystis* sp. (LEANCYA 5, 13, 17, 20, 21) and *Synechococcus* sp. (LEANCYA 11, 16, 18, 19)	primary rat hepatocytes and HL-60 cells	−	[100]
*Cyanobium* sp. (LEGE 06098, 06134, 07175, 07186, 06113, 06137, 006097, 06139)	human cancer cell lines	−	[101]
*Synechococcus nidulans* (LEGE 07171)	human cancer cell lines	−	[101]
*Synechococcus* sp. (LEGE 07172, 06005, 06026),	human cancer cell lines	−	[101]
*Synechocystis salina* (LEGE 06099, 06155, 07173)	human cancer cell lines	−	[101]
endosymbiotic *Synechococcus*-like cyanobacterium	human leukemia cell line HL-60	−	[90]
endosymbiotic *Synechococcus*-like cyanobacterium	*Escherichia coli*, *Staphylococcus aureus*, *Saccharomyces cerevisiae*, *Mucor hiemalis*, *Ruegeria atlantica*	0	[90]
*Synechococcus leopoliensis* (Utex 625)	*Staphylococcus aureus*	−	[102]
*Synechocystis* sp. (LEANCYA 1, 5, 13, 17, 20, 21) and *Synechococcus* sp. (LEANCYA 7, 10, 11, 16, 18, 19)	*Artemia salina*	−	[103]
*Synechocystis* sp. (LEANCYA 1, 5, 13, 17, 20, 21) and *Synechococcus* sp. (LEANCYA 7, 10, 11, 16, 18, 19)	*Brachionus plicatillis*	0	[103]
*Synechocystis* sp. (LEANCYA 1, 5, 17, 20, 21) and *Synechococcus* sp. (LEANCYA 7, 11, 16, 18, 19)	*Paracentrotus lividus*	−	[103]
*Synechocystis* sp. (LEANCYA 21) and *Synechococcus* sp. (LEANCYA 16)	*Mytilus galloprovincialis*	−	[103]
*Cyanobium* sp. (LEGE 06008, LEGE 06011 and LEGE 06015)	*Artemia salina*	−	[104]
*Synechococcus* sp. (LEGE 06005)	*Artemia salina*	−	[104]
*Cyanobium* sp. (LEGE 06098, 06134, 06139, 07175, 07186)	*Artemia salina*	0	[105]
*Cyanobium* sp. (LEGE 06098, 06134, 06139, 07175, 07186)	eggs of the sea urchin *Paracentrotus lividus*	−	[105]
*Cyanobium* sp. (LEGE 06098, 06134, 06139, 07175, 07186)	*Pseudomonas* sp.	−	[105]
*Cyanobium* sp. (LEGE 06098, 06134, 06139, 07175, 07186)	*Nannochloropsis* sp.	−	[105]
*Synechococcus* sp. (CC9311)	*Embiotoca jacksoni*	−	[106]
*Synechococcus* sp. (CC9902)	*Embiotoca jacksoni*	0	[106]
*Cyanobacterium* sp., *Synechococcus* sp., *Synechocystis* sp.	liver, kidney, small intestine and lungs of mice	−	[75]
